# Developmental Origins of Pediatric Obesity

**DOI:** 10.1155/2012/309863

**Published:** 2012-08-15

**Authors:** Sheila Gahagan, Ricardo Uauy, Tessa J. Roseboom

**Affiliations:** ^1^Division of Academic General Pediatrics, Child Development and Community Health, UC San Diego, CA 92093-0927, USA; ^2^Center for Human Growth and Development, University of Michigan, Ann Arbor, MI 48109-5406, USA; ^3^Institute of Nutrition and Food Technology (INTA), University of Chile, El Líbano 5524, 138-11 Santiago, Chile; ^4^Department of Pediatrics, Catholic University of Chile, Avenida Libertador General Bernardo O'Higgins 340, Santiago, Chile; ^5^Department of Clinical Epidemiology, Biostatistics and Bioinformatics and Department of Obstetrics and Gynecology, Amsterdam Academic Medical Center, 1105 AZ Amsterdam, The Netherlands

Obesity is a global pandemic, with rates increasing in both the developed and developing world. Childhood obesity is a significant concern as it has negative consequences for other childhood morbidities and is often associated with adult obesity, diabetes, and cardiovascular disease.

There is an increasing body of evidence to suggest that obesity can develop in relation to early life events. This special issue focuses on new research into the developmental origins of childhood obesity. The early determinants of obesity are elegantly described in a paper in this special issue by K. E. Rhee and colleagues. They describe that genes, epigenetics, and the *in utero* environment can affect whether or not a child develops obesity. In general, obese parents are more likely to have obese children, and genetic makeup plays a role. The tremendous increase in obesity prevalence in the past decades, however, cannot be explained by genetic change alone. The change in prevalence over time has simply been too fast. The environment has changed and it is likely that a combination of genetic susceptibility to obesity with an obesogenic environment explains a large part of the rise in pediatric obesity. This gene-environment interaction may also explain individual variations in obesity development, that is, why one sedentary child eating a high-fat diet becomes obese and another child who eats similarly does not. The developmental origins hypothesis, which proposes that suboptimal conditions in early life can alter gene expression and lead to lifelong changes in the body's organs and tissues, could help to explain such enigmas. A paper by J. G. Eriksson and colleagues, in this special issue, describes an example of such a gene-environment interaction. In the Helsinki birth cohort study, they found that placental size and shape were associated with obesity, but that the associations depended on genetic makeup. A large placenta was associated with overweight status and high percent body fat in a subset of men and women whose mothers were tall and who carried the Pro12Pro genotype of the PPAR*γ* 2 gene. This suggests that there is interplay between nutritional factors and genes at the placental level that affects later risk for obesity.

Two papers in this special issue describe modifiable maternal factors associated with offspring risk for obesity. Using data on over 40 thousand mother-child pairs from the Danish National Birth Cohort, the study by C. S. Andersen et al. describes how maternal exercise during pregnancy relates to offspring BMI at age 7 years. Maternal recreational exercise during early and late pregnancy was related to offspring BMI and obesity risk when they were 7 years old. While recreational exercise during pregnancy was inversely related to offspring BMI, the associations were largely explained by lifestyle factors such as smoking, socioeconomic status, and maternal prepregnancy BMI. Additionally, exercise intensity or changes in exercise habits during pregnancy were not related to the children's BMI or obesity risk. The other paper that addresses the association between modifiable factors during pregnancy and offspring's obesity underscores the impact of smoking. Using analyses of the NHANES data, S. E. Messiah et al. showed that smoking during pregnancy was associated with higher risk of obesity in the offspring. Additionally, breastfeeding was protective against early childhood obesity. These 2 papers add additional evidence supporting the importance of ongoing maternal exercise, refraining from smoking and breastfeeding as strategies to reduce risk for offspring obesity.

Also included are 2 papers assessing the risk of higher weight gain over the first 3 months of life on later health outcomes. From a population-based cohort of healthy newborns in the Netherlands, A. M. V. Evelein et al. found that increased weight for length gain during the first 3 months was associated with higher BMI and subcutaneous abdominal fat 5 years later. Their finding was independent of birth weight and therefore of fetal weight gain. This suggests that accretion of fat over the first months of life is associated with body fat later in childhood. Also assessing the role of weight gain over the first 3 months of life, K. Khuc et al. found an association with signs of the metabolic syndrome at 16 years in a longitudinal cohort from Santiago, Chile. They found that early infancy weight gain was significantly associated with a higher metabolic syndrome risk score in adolescence. On the other hand, exclusive breastfeeding for 90 days or more was protective for signs of the metabolic syndrome. While the contexts of these 2 studies are markedly different, both find that more rapid weight gain in the first months of life is associated with risk for potentially adverse outcomes, years later.

D. E. Kang Sim and colleagues assessed determinants of infant growth. In a cohort of low- to middle-income Chilean infants, they assessed the relationship of psychosocial factors to infant length and weight gain, finding that higher SES was directly related to length growth over the first year, consistent with the well-known association between social status and height. Higher SES was also related to weight gain but indirectly through family factors including family composition, the physical environment, and maternal warmth. All of the factors associated with more rapid weight gain were markers of good nurturing. Failure to thrive (undernutrition) was nonexistent in this cohort. In an era of overnutrition, it is sobering to grasp that good parenting may create risk for the development of obesity and related cardiovascular and metabolic risk.

Nutritional status outside of infancy can also create risk for obesity. The paper by A. F. M. van Abeelen et al. on the role of exposure to various degrees of undernutrition during the Dutch Hunger Winter demonstrates that a limited period of severe childhood undernutrition, followed by adequate nutrition was associated with higher BMI and waist circumference, measured approximately 50 years later, in exposed women compared to those who were relatively unexposed. Furthermore, the risk for overweight or obesity was significantly increased in women who had been severely exposed to famine between birth and 9 years compared to women in the same age cohort who were relatively unexposed.

The evidence presented in this issue supports the notion that obesity prevention should start *before conception and extend at least through the first 1 to 2 years of life*. Acting early can change lifetime predisposition for obesity not only effectively but also cost effectively. Research on the developmental origins should now move beyond the descriptive stage to a more proactive stage; addressing the controlled evaluation of preventive strategies based on recent findings such as those reported in this special issue of the International Journal of Pediatrics. We are faced with pressing questions related to bringing evidence from epidemiological and clinical studies to clinical and community-based interventions. On the one hand, how do we translate these findings to actual recommendations for health professionals, especially obstetricians and pediatric practitioners? And, on the other, how do we effectively implement evidence-based recommendations on a sufficiently large-scale to have a measurable impact? We must start from a firm base of normative data on normal growth in utero and after birth. These standards should be based on longitudinal follow-up of large cohorts, with defined normative entry criteria, living in environments that promote and support normal growth. The soon-to-be released international fetal growth curves from the intergrowth study will provide normative longitudinal data for *in utero* fetal growth for the first time [1]. The multicentre growth reference study (WHO growth standards) for infants and children up to age 5 years of age, based on infants predominantly breast fed for the first 6 months of life, are the best available growth standards for now [2]. Eventually optimal growth should be defined related to long-term health outcomes.

It is crucial to develop and test new clinical models. We must convey the need to prepare for pregnancy as a first vital step for the critical stages of implantation and placentation, which support normal embryonic development. This major change in clinical care requires the establishment of a standard preconceptional reproductive health visit. This visit would allow clinicians to focus on normalizing prepregnancy weight and ensuring micronutrient sufficiency as a first step to optimal preconceptual health. Prior to pregnancy, it is important to insure that women have nutritious diets that prevent excess adiposity and supply appropriate doses of folate, iron, and zinc. At the current time, we do not know the best nutritional strategies for pregnancy in overweight and obese women. Recent Institute of Medicine (IOM) recommendations for weight gain during pregnancy (tailored to prepregnancy weight status) provide the basis for developing clinical guidelines to achieve these goals [3]. Furthermore, in developing countries, guidelines for nutrition in pregnancy should consider maternal height as it signals the mother's past nutritional status. It is important to be mindful of the risk of excess nutrition especially in those who experienced inadequate nutrition in early life.

In most settings, clinical practices are left for each practitioner to decide. For example, we lack good evidence for nutritional guidance during the transitional feeding period for infants from 6 to 12 months. In addition, there is also almost no information on how to achieve physical activity recommendations during pregnancy and infancy, and even less, on how to effectively transmit these messages to best promote and support behavior changes at the population level. Health professionals are faced with the challenge of providing diet and physical activity recommendations based on their own experience and criteria. Normative growth monitoring and recommended actions to be taken when deviations from the norm occur are seldom standardized. Appropriate responses to questions such as, “When is weight gain during pregnancy a matter of concern?” or “When should complementary foods or formula supplementation be provided for an infant who is failing to grow?” remain unspecified in most primary health settings worldwide. More importantly, current decision-making algorithms are almost nonexistent.

If we want to maximize the results of obesity preventive actions, we urgently need clinic-, community- and health system-based intervention trials to address these issues. Formative research is also required to improve delivery of strategies with known effectiveness. Therein lies the greatest opportunity for moving from efficacy to effectiveness and public health impact. Most countries have maternal and infant nutrition health and welfare programs in place, providing unique opportunities for testing and evaluating these recommended actions. Effective interventions could then be implemented at scale; thus expanding the impact to the health of entire populations. Early life should now be presented not only as a very attractive window for effective prevention of undernutrition [4] but also for obesity preventive actions. In fact, we should focus on the time from before conception to 2 years postnatal life as critical to the prevention of malnutrition in all its forms [5] and the promotion of “healthy growth.” We can begin with recent guidance regarding growth monitoring during pregnancy (IOM recommendations) and infancy (WHO standards), and continue to promote breastfeeding as the best feeding mode for the first 6 months of life.

While the latest systematic Cochrane review finds limited effectiveness from controlled interventions on prevention of childhood obesity [6], there is reason for optimism. The Healthy Beginnings Trial from Australia, a recent large randomized-controlled trial of a home-based early life intervention delivered by trained community nurses, resulted in improvements in TV viewing and feeding/eating behaviors and a significant reduction in mean BMI at 2 years [7]. The goal for new clinical practices is to promote appropriate adiposity for life-long health. In order to do this, we need new clinical approaches as outlined but this will not be sufficient. All of these efforts will be futile if we fail to build environments that facilitate and sustain changes aimed at healthy nutrition and physically active lifestyles [8]. The combination of promoting optimal weight gain from the time of conception continuing into childhood with healthy environments will ensure improved long-term health for our children and generations to come.
